# Functional Characterization and Cellular Dynamics of the CDC-42 – RAC – CDC-24 Module in *Neurospora crassa*

**DOI:** 10.1371/journal.pone.0027148

**Published:** 2011-11-07

**Authors:** Cynthia L. Araujo-Palomares, Corinna Richthammer, Stephan Seiler, Ernestina Castro-Longoria

**Affiliations:** 1 Department of Microbiology, Center for Scientific Research and Higher Education of Ensenada (CICESE), Ensenada Baja California, México; 2 Institut für Mikrobiologie und Genetik, Universität Göttingen, Göttingen, Germany; Cinvestav, Mexico

## Abstract

Rho-type GTPases are key regulators that control eukaryotic cell polarity, but their role in fungal morphogenesis is only beginning to emerge. In this study, we investigate the role of the CDC-42 – RAC – CDC-24 module in *Neurospora crassa*. *rac* and *cdc-42* deletion mutants are viable, but generate highly compact colonies with severe morphological defects. Double mutants carrying conditional and loss of function alleles of *rac* and *cdc-42* are lethal, indicating that both GTPases share at least one common essential function. The defects of the GTPase mutants are phenocopied by deletion and conditional alleles of the guanine exchange factor (GEF) *cdc-24*, and *in vitro* GDP-GTP exchange assays identify CDC-24 as specific GEF for both CDC-42 and RAC. *In vivo* confocal microscopy shows that this module is organized as membrane-associated cap that covers the hyphal apex. However, the specific localization patterns of the three proteins are distinct, indicating different functions of RAC and CDC-42 within the hyphal tip. CDC-42 localized as confined apical membrane-associated crescent, while RAC labeled a membrane-associated ring excluding the region labeled by CDC42. The GEF CDC-24 occupied a strategic position, localizing as broad apical membrane-associated crescent and in the apical cytosol excluding the Spitzenkörper. RAC and CDC-42 also display distinct localization patterns during branch initiation and germ tube formation, with CDC-42 accumulating at the plasma membrane before RAC. Together with the distinct cellular defects of *rac* and *cdc-42* mutants, these localizations suggest that CDC-42 is more important for polarity establishment, while the primary function of RAC may be maintaining polarity. In summary, this study identifies CDC-24 as essential regulator for RAC and CDC-42 that have common and distinct functions during polarity establishment and maintenance of cell polarity in *N. crassa*.

## Introduction

Rho GTPases are small G proteins of the Ras superfamily and function as molecular switches that activate a variety of effector proteins when in the GTP-bound state and return to inactivity upon hydrolysis of GTP. They play key roles in multiple signal transduction pathways and regulate fundamental cellular processes, including cell migration, cell cycle progression and cell polarity [1], [2]. Rho guanine nucleotide exchange factors (RhoGEFs) and Rho GTPase-activating proteins (RhoGAPs) enhance nucleotide binding and hydrolysis of Rho GTPases, respectively, and are increasingly acknowledged as crucial determinants of spatio-temporal Rho signaling activity [3].

While the unicellular yeasts *Saccharomyces cerevisiae* and *Schizosaccharomyces pombe* are the paradigms for polarized growth, many members of the fungal kingdom, among them important human pathogens, are distinguished from their well-studied yeast relatives by their ability to grow in a filamentous mode, leading to the formation of highly elongated hyphae. Knowledge about the molecular mechanisms underlying this extreme form of polarized extension is only slowly beginning to accumulate, and Rho GTPases and their regulators play an essential role in hyphal morphogenesis and development [4]–[9].

The most obvious distinction between the Rho repertoires in yeasts and filamentous fungi is the presence of a Rac homologue in the latter organisms only. Rac is considered the founding member of the Rho GTPase family, from which the closely related Cdc42 and the more distantly related Rho proteins descended, a process associated with concomitant specialization of function [10]. In yeasts, Rac was probably lost later and its roles taken over by Cdc42. This scenario would explain why S. *cerevisiae* and *S. pombe* cells devoid of Cdc42 are not viable [11], [12], while its depletion in filamentous fungi is not lethal, but becomes so upon simultaneous disruption of the Rac-encoding gene [13], [14]. In mammalian systems, Rac and Cdc42 are best known for their control of different actin-based cell projections involved in cell motility. While Rac is the main regulator of lamellipodia formation, Cdc42 is required for formation of the slender filopodia, and the two GTPases appear to regulate both unique and shared effector proteins [15], [16]. A similar tendency to employ Rac and Cdc42 for both overlapping and distinct morphogenetic functions is also observed in filamentous fungi, although it is also becoming clear that the degree of specialization between the two GTPases and their relative contributions to hyphal growth can vary widely between different species [17].

For instance, in *Candida albicans*, a dimorphic ascomycete and opportunistic human pathogen, deletion of rac1 does not interfere with viability, but Cdc42 is an essential gene. Moreover, Rac1 and Cdc42 have distinct roles in hyphal growth triggered by different stimuli and cannot substitute for each other [18], [19]. Rac1 and its GEF Dck1 are required for matrix-induced filamentous growth and appear to be involved in cell wall integrity [19], [20], [21]. On the other hand, specific regulation of Cdc42 and its essential GEF Cdc24 allows serum-induced filament formation [18], [22]. In contrast, in the basidiomycete *Ustilago maydis* Rac1 plays the prominent role during hyphal growth, while deletion of Cdc42 does not affect filament formation. In contrast, Cdc42, but not Rac, is essential for cell separation of the yeast cells after cytokinesis and triggers the formation of the secondary septum [13]. Thus, the roles of Cdc42 and Rac1 have strongly diverged, and consistently the two GTPases cannot substitute for each other [23]. Nevertheless, despite the high degree of specialization, the two GTPases must have retained at least one common essential function, as evident in the synthetically lethal effect of their combined depletion [13].

RacA and Cdc42 of the filamentous ascomycete *Aspergillus nidulans* are proposed to share a function in establishing the primary axis of polarity. Cdc42 appears solely responsible for maintaining directed elongation and regulating subsequent polarization events for lateral branch formation while RacA appears to play a prominent role in asexual development [14]. Only recently, it has been shown that in *Aspergillus niger*, which is a close relative of *A. nidulans*, RacA has a prominent role in regulating actin polarization and hyphal growth, especially maintenance of established polarity axes, while the Cdc42-homologue CftA appears largely dispensable [24].

Initial hints for the involvement of Rho GTPases for hyphal morphogenesis in the filamentous ascomycete *Neurospora crassa* came from a large-scale screen for conditional mutants defective in cell polarity that identified conditional mutants in the RHO1-specific GAP *lrg-1* and the GEF *cdc-24* [25], [26]. In this work, we investigate the requirement of CDC-42 and RAC during polarization and growth of *N. crassa* and explored their common regulation through the GEF CDC-24.

## Materials and Methods

### Strains, media and growth conditions

*N. crassa* and bacterial strains used in this study are listed in Table 1. General genetic procedures and media for *N. crassa* are available through the Fungal Genetics Stock Center (www.fgsc.net; [27]). Fungal strains were routinely grown at 28°C on Vogel's Minimal Medium (VMM) supplemented with 1.5% (w/v) sucrose as the carbon source and solidified with 1.5% (w/v) agar. Stock solutions of cytochalasin A and benomyl (Sigma-Aldrich, St. Louis, Mo) were prepared in 100% ethanol at 10 mg/ml. Working solutions of cytochalasin and benomyl were prepared according to [28] at 2.5 µg/ml and 1.0 µg/ml respectively. A drop of the drug was placed on a coverslip and the block of agar containing mycelium was placed in contact with the inhibitor solution and scanned under confocal microscopy after 5 min of exposure. For auxotrophic strains, 0.5 mg/ml histidine was added to VMM [29]. Transformation of *N. crassa* macroconidia was carried out by electroporation as previously described [30]. *N. crassa* crosses were carried out on synthetic crossing medium [31]. Transformants showing robust consistent fluorescence were selected and back-crossed to obtain homokaryotic strains. Mycelium for DNA extraction was grown for 7 days on VMM liquid medium with no shaking and no light, filtered, submerged in liquid nitrogen and lyophilized. For genomic DNA extraction of *N. crassa*, we used the DNeasy Plant extraction Kit (Qiagen, Inc.). Temperature-sensitive strains of *cdc-42* and *rac* were created by applying RIP (repeat induced point mutation) mutagenesis [32]. Briefly, *N. crassa his-3* was transformed with 1.5 kb fragments covering *rac* and *cdc-42* coding sequence and 350 bp and 200 bp 5′ and 3′, respectively, cloned into vector pBM61 [30]. These strains were mated with wild type and the resulting progeny screened for conditional growth defects according to the procedure described in [25].

**Table 1:** *Neurospora crassa* strains used in this study.

Strain	Genotype	Source
*wild type* mat A	*74-OR23-1A*	*FGSC1* 1 *#987*
*wild type* mat a	*ORS-SL6a*	FGSC #4200
*wild type* mat a	*74-OR8-1a*	FGSC #988
*mus-51* mat A	*Δmus-51::bar^+^; his-3^−^*	FGSC #9717
*cdc-24(10-19)*	*cdc-24(F254S)*	[25]
*cdc-24(19-3)*	*cdc-24(L444S)*	[25]
*cdc-24(24-21)*	*cdc-24(Q264R)*	[25]
*rac(7-1)*	*rac(G16S)*	this study
*cdc-42(18-4)*	*cdc-42(D16N, L22P, A161T, D172N)*	this study
*rac(7-1);cdc-42(18-4)*	*rac(G16S);cdc-42(D16N, L22P, A161T, D172N)*	this study
*Δrac*	Δ*rac::hph^R^ a*	FGSC #11525
*Δcdc-42*	Δ*cdc42::hph^R^ a*	FGSC #15833
*Δcdc-24*	Δ*cdc-24::hph^R^ + cdc-24^+^ a*	FGSC #11721
*yfp-cdc-42*	*pgpd-yfp-cdc-42::his-3*; Δ*cdc-42::hph^R^*	this study
*yfp-rac*	*pgpd-yfp-rac::his-3*; Δ*rac::hph^R^*	this study
*gfp-cdc-24*	*his-3^+^::Pccg-1::gfp^+^::cdc-24^+^ A*	this study

1 Fungal genetics stock center.

### Plasmid construction of fluorophore-Rho fusion proteins

For creation of pPgpdYFP_Rac and pPgpdYFP_Cdc42, respectively, *rac* (NCU02160) and *cdc42* (NCU06454) were amplified from genomic DNA using primer combinations SB_rac_5_BglII/SB_rac_3_EcoRI and SB_cdc42_5_BglII/SB_cdc42_3_EcoRI, respectively, and inserted via BglII/EcoRI sites into pPgpdYFP, which was designed to allow expression of N-terminally yellow fluorescent protein (YFP)-tagged proteins under the control of the *A. nidulans gpdA* promoter. The promoter was amplified from plasmid pEHN1-nat [33] using primers CoS_Pgpd_3/_4, while *yfp* was amplified from pYFP [34] using primers CoS_YFP_1/CoS_YFPC_2MCS. The two fragments were subjected to fusion PCR with primer pair CoS_Pgpd_3/CoS_YFPC_2MCS. The resulting amplification product was cleaved with ApaI/NotI and inserted into pYFP from which the *ccg-1* promoter and *yfp* gene had been released by digestion with the same enzymes. For creation of pCAP24.3GFP_Cdc24, *cdc24* (NCU06067.4) was amplified from genomic DNA using primer combinations cdc24_5_SpeI/cdc24_3_PacI and inserted via SpeI/PacI sites into pRM-12GFP (Mouriño-Pérez, unpublished). Plasmids and oligonucleutides used or generated in this study are listed in Tables S1 and S2, respectively.

### GEF assays

cDNA encoding wild type and mutant versions of RhoGEF and PH domain regions of CDC24 (NCU06067; aa 204–544) were amplified using primers NV_CDC24_5/_6. SalI/NotI sites were used for ligation with pNV72 to produce pMalc2xL_CDC24GEFPH and its respective mutant analogues. The *N. crassa* Rho GTPases and RhoGEF domain constructs were expressed as fusion proteins with an N-terminal maltose binding protein (MBP) tag. For fusion protein purification (modified from [26], [35]), LB+ medium (1% NaCl, 0.8% yeast extract, 1.8% peptone, 2% glucose) was inoculated to an OD_600_ of 0.1 from an overnight culture of Rosetta2(DE3) *E. coli* cells transformed with the respective pNV72-derived plasmid. Cultures were grown shaking at 20°C to an OD_600_ of 0.45, and fusion protein expression was induced by addition of isopropyl β-D-thiogalactopyranoside to 0.2 mM for 2 hours. Cells were disrupted by ultrasonication using a Sonopuls HD 2070 ultrasonicator (Bandelin GmbH & Co. KG, Germany) in lysis buffer (50 mM Tris, pH 7.4, 125 mM NaCl, 5 mM MgCl_2_, 10% glycerol, 0.02% NP-40, 2 mM DTT, 1 mM PMSF, 0.35 mg/ml benzamidine, 10 µM GTP) and cleared lysates incubated on a rotating wheel at 4°C with pre-equilibrated Amylose Resin (New England Biolabs, USA) for one hour. The resin was washed twice with washing buffer (lysis buffer with 250 mM NaCl) before elution with elution buffer (50 mM Tris, pH 7.4, 200 mM NaCl, 5 mM MgCl_2_, 10% glycerol, 0.02% Nonidet-P40, 2 mM DTT, 20 mM maltose). Total protein concentration of the eluate was determined with bovine serum albumin standard solutions as a reference and using Roti®-Quant (Carl Roth GmbH+Co. KG, Germany) and a Tecan Infinite® M200 microplate reader equipped with Magellan™ software (version 6; both Tecan Group Ltd., Switzerland).

Intrinsic and GEF-stimulated *in vitro* Rho GTPase nucleotide exchange activity was measured using the fluorescent guanine nucleotide analogue mant-GDP (2′/3′-O-(N′- methylanthraniloyl)-GD), which exhibits markedly increased emission intensity upon binding to a protein [36]. Assay procedures were modified from [35], [37]. Reaction mixtures contained 0.1 µM mant-GDP, 10 mM NaH_2_PO_4_/K_2_HPO_4_, pH 7.5, 1.2 µM MBP-Rho GTPase and/or 0.8 µM MBP-GEF in reaction buffer (30 mM Tris, pH 7.4, 5 mM MgCl_2_, 3 mM DTT); exchange reactions were started by addition of mant-GDP and, where applicable, GEF protein. Changes in fluorescence intensity (λ_exc_ = 356 nm, λ_em_ = 448 nm; [arbitrary units]) were monitored using the Tecan Infinite® M200 plate reader at 21°C over 24 minutes. Measured data were corrected for background signals for each time point, and linear slope of fluorescence intensity over time was calculated and averaged for technical replicates. Relative values were calculated by normalizing to the respective value of intrinsic exchange activity of each Rho GTPase, which was set to 100%.

### Live-cell imaging

For the analysis of colonial and hyphal morphology, an Olympus SZX16 (Olympus, Japan) stereomicroscope equipped with an Olympus SDF PLAPO 1xPF objective was used; photos were captured with an Olympus ColorView III camera operated by the program Cell^D^ analySIS Image Processing (Olympus SoftImaging Solutions GmbH, Germany). Higher resolution images of *N. crassa* hyphae were obtained using the inverted agar block method [38] on an inverted Zeiss Laser Scanning Confocal Microscope LSM-510 META provided with an Argon-2 ion and a He/Ne1 lasers well suited to detect GFP and YFP Abs/Em 488/515–530 nm. A Plan Apochromat X100/1.4 oil immersion objective was used. A photomultiplier module allowed us to combine fluorescence with phase-contrast to provide simultaneous view of the fluorescently labeled proteins and the entire cell. Confocal images were captured using LSM-510 software (version 3.2; Carl Zeiss, Germany) and evaluated with an LSM-510 Image Examiner (version 3.2). Some of the image series were converted into animation movies using the same software. Samples were stained and incubated with 2.5 µM of FM4-64 for 10 min (Molecular probes, Eugene, OR) and subsequently analyzed under confocal microscopy using an Argon-2 laser, Abs/Ems 514/670 nm.

## Results

### The coordinated activity of RAC and CDC-42 is required for cell polarization, the integrity of the Spitzenkörper and hyphal growth of *N. crassa*

In order to dissect the functions of RAC and CDC-42 for hyphal growth in *N. crassa*, we generated conditional mutants using an *in vivo* mutagenesis approach. RIP (repeat induced point mutation) is a unique method that allows the introduction of point mutations in *N. crassa* genes as part of a defense mechanism of *N. crassa* by inactivating duplicated sequences when strains carrying repeat sequences are sent through a cross [32]. By visually screening ca. 10.000 progenies of crosses of wild type with strains carrying duplicated *rac* or *cdc-42* genes for temperature-sensitive phenotypes, we identified one strain in the progeny of each cross that displayed conditional growth defects. Sequencing of the *rac(7-1)*, *cdc-42(18-4)*, coding regions amplified from genomic DNA of these mutants revealed several silent mutations, but also RIP-specific mutations that translated to one and four amino acid substitutions of highly conserved positions, respectively (Figure S1). An alignment of *N. crassa* RAC and CDC-42 with homologues from other fungi revealed that the substitutions are all located at conserved positions.

**Figure 1:**
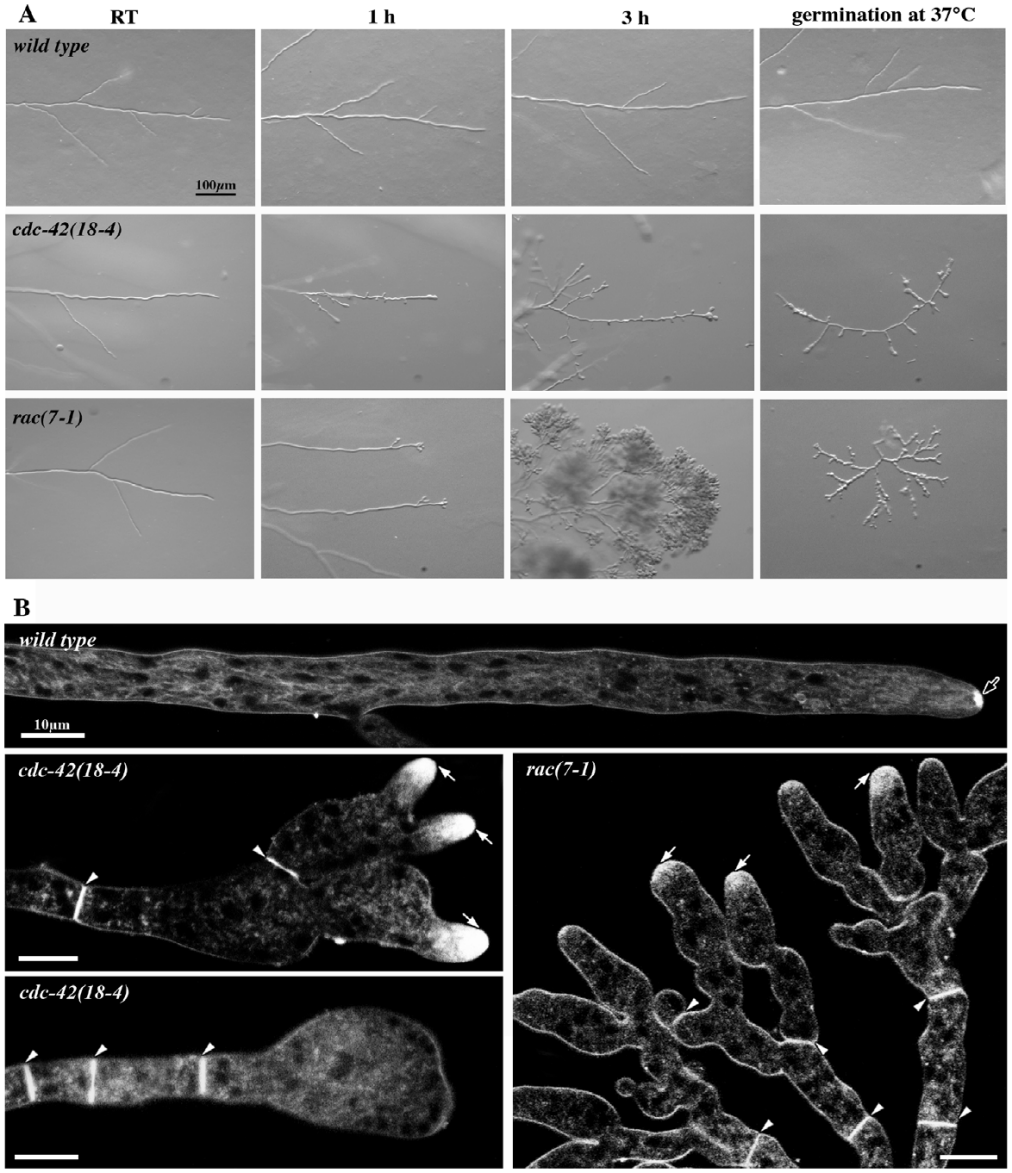
Conditional *rac* and *cdc-42* mutants reveal cell polarity defects and aberrant hyphal morphogenesis. (**A**) Stereomicroscopic analysis of *N. crassa* wild type, *cdc42(18-4)* and *rac(7-1)* grown at permissive conditions and shifted to 37°C for the indicated time or germinated at restrictive temperature. (**B**) Higher magnification and staining with FM4-64 revealed severe morphological defects of *cdc42(18-4)* and *rac(7-1)* grown at 37°C. Note that the dye accumulates within the apical region of the hyphal tip (white arrows), but a typical Spk (black arrow in wild type) is not formed. Arrowheads mark septa in the conditional strains.

When grown at permissive conditions (≤32°C), *rac(7-1)* and *cdc-42(18-4)* exhibited normal cell morphology, albeit slightly reduced growth rates. Shifting cultures to 37°C, however, quickly lead to pronounced morphological aberrancies (Figure 1 A). As control, wild type was cultured under the same experimental conditions and no morphological changes were detected. Labeling these mutants with the vital dye FM4-64 revealed that some hyphae still displayed accumulation of the colorant at the apex of the new branches, but the typical Spitzenkörper (Spk) observed in wild type was not present in the two mutants at restrictive conditions (Figure 1 B). *cdc-42(18-4)* hyphae shifted to restrictive temperatures exhibited some apical branching, but its most prominent defects, however, were the loss of apical polarity, the frequent generation of subapical branches and swelling of most hyphal tips. Pronounced apical hyperbranching was observed in *rac(7-1)* within 30 min of transfer to restrictive conditions. Many of these new tips grew initially in an apolar manner, but resumed some polarity after prolonged incubation at 37°C, resulting in knobby tree-like clusters of hyphae at the edge of the highly compact colony. Nevertheless, the strong polarity defect in both conditional mutants didn't affect all hyphal tips and allowed the formation of compact colonies with highly reduced extension rates even at restrictive conditions.

**Figure 2:**
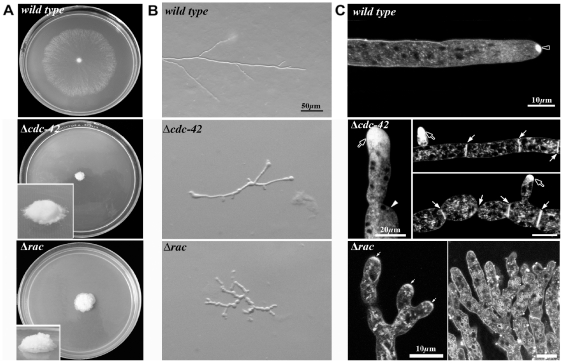
Δ*cdc42* and Δ*rac* strains are viable, but cell morphogenesis is strongly impaired. (**A**) Strains incubated at ambient conditions, upper panel shows growth of wild type after 24 h of incubation and lower panel depicts the small and compact Δ*cdc42* and Δ*rac* colonies formed within 48 h. Stereoscopic analysis (**B**) and higher resolution imaging of Δ*cdc42* and Δ*rac* stained with FM4-64 (**C**) reveal strong polarity defects. Note the FM4-64 accumulation at the apical region of the tip (black arrows in Δ*cdc42*; small white arrows in Δ*rac*) but the absence of a typical Spk (black arrowhead in wild type). White arrowhead point out at swollen tip and site of apolar growth, white arrows indicate septa.

We also isolated several clones with compact morphologies from the *rac* and *cdc-42* crosses that did not show conditional defects. Sequencing the GTPase genes of these mutants revealed the repeated generation of in frame stop codons within the *rac* and *cdc-42* coding region, suggesting that these mutants are loss of function alleles of the two GTPases (data not shown). Because clear deletion strains were available form the *Neurospora* genome project [39], [40], we focused on the further characterization of these strains instead of the loss of function mutants isolated in the RIP approach. Colonies of *Δcdc-42* and *Δrac* showed severe growth defects, resulting in a very compact colony morphology, in contrast to the typical spreading growth of wild type (Figure 2 A). They also showed irregular growth generating distorted hyphae as a consequence of temporal loss of polarity and the periodical re-initiation of polar growth at the site of swollen tips (Figure 2 B). When stained with FM4-64, *Δcdc-42* displayed a bright accumulation of the dye at the apical-subapical area without generating a defined Spk as clearly observed in wild type (Figure 2 C). Septa were also abundant and generated near the apical zone, resulting in cell compartments of reduced length (Figure 2 C). *Δrac* was typified by its production of profuse apical branches, resulting in ramification of the compact colony. Accumulation of FM4-64 in the apical tip region was lower when compared with hyphal tips of *Δcdc-42*. These observations corroborate that RAC and CDC-42 are critical components for polarity maintenance and are required for Spk assembly.

**Figure 3:**
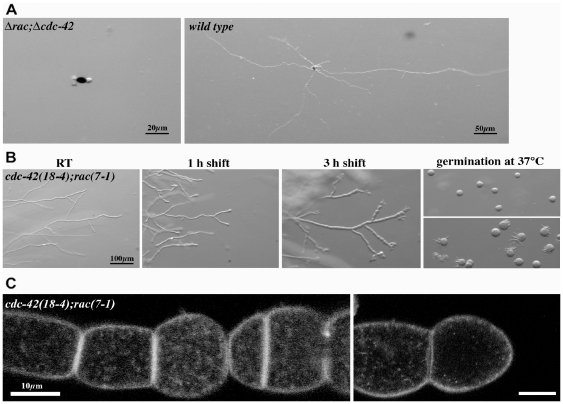
*rac* and *cdc-24* mutants are synthetically lethal. (**A**) A Δ*rac*;Δ*cdc-42* double mutant is lethal and ascospores plated on selective medium germinate apolarly and die while the colony development initiates after germination of wild type ascospores under the same conditions. (**B**) Stereomicroscopic analysis of a conditional *cdc42(18-4)*;*rac(7-1)* strain grown at permissive conditions and shifted to 37°C for the indicated time or germinated at restrictive temperature. Note that re-polarization of swollen conidia after downshift to permissive conditions occurs within 15 min, resulting in the formation of multiple germ tubes generated primarily on one side of the spore. The hyphal morphology of a wild type control grown under these conditions is shown in Figure 1. (**C**) Higher magnification of mature hyphae shifted to restrictive conditions for several h and stained with FM4-64 reveal hyperseptation and chains of swollen cells.

We were unable to obtain viable Δ*rac*;Δ*cdc-42* strains, but the frequent occurrence of apolarly germinating ascospores obtained from Δ*rac*×Δ*cdc-42* crosses suggested lethality of the double mutants (Figure 3 A). We tested this hypothesis by generating conditional *rac(7-1);cdc-42(18-4)* double mutant. In accordance with the proposed requirement of at least RAC or CDC-42 function for viability, this strain displayed strong synthetic growth and polarity defects upon transfer to 37°C (Figure 3 B). We observed pronounced apical hyperbranching, concomitant swelling of apical and subapical hyphal compartments and the increased formation of septa. After 3 h of incubation cell polarity was completely lost, and the swollen compartments lysed and died after prolonged incubation at restrictive conditions. This was confirmed by confocal imaging using FM4-64, which also revealed that the typical hyphal organization was lost (Figure 3 C). Germination of *cdc-42(18-4);rac(7-1)* conidia at restrictive temperature did not produce viable germlings, but only cells grew isotropically before they lysed. Shifting these isotropically swollen cells back to permissive conditions resulted in the fast generation of multiple germ tubes (Figure 3 B). Interestingly these tubes emerged primarily on one side of the spore, suggesting that signals required for polarity establishment are not confined to a single spot but to a wide region of the cell. In summary, these phenotypic characteristics indicate a common, essential function of the two GTPases for establishment and maintenance of cell polarity in additional to individual, but non-essential functions during hyphal morphogenesis.

### CDC-24 functions as exchange factor and activator of RAC and CDC-42

**Figure 4:**
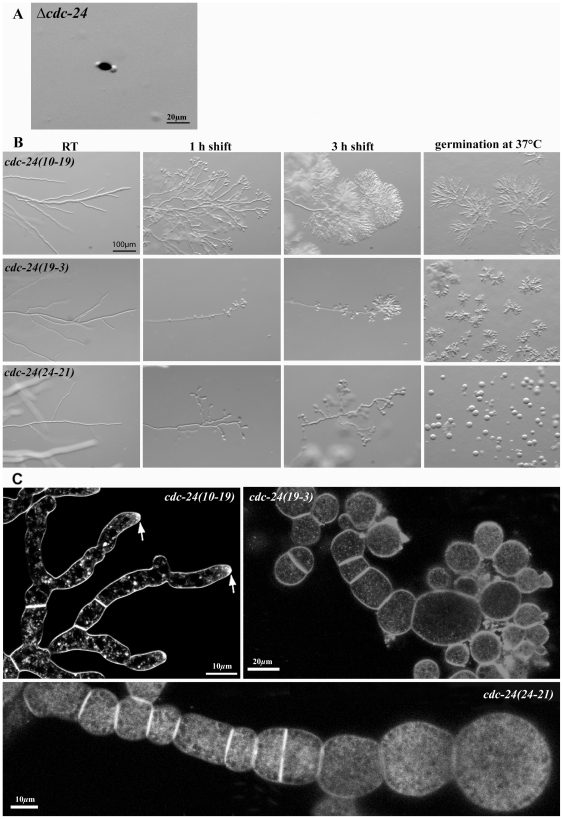
*cdc-24* is essential for viability and conditional alleles phenocopy defects observed in *rac* and *cdc-42* mutants. (**A**) Deletion of *cdc-24* is lethal and ascospores plated on selective medium germinate apolarly and die. (**B**) Stereoscopic analysis, of conditional *cdc24(10-19)*, *cdc24(19-3)* and *cdc24(24-21)* strains of *N. crassa* grown at permissive conditions and shifted to 37°C for the indicated time or germinated at restrictive temperature. Defects range from apical hyperbranching in the weak *cdc24(10-19)* allele to complete failure to polarize in *cdc24(24-21)*. The hyphal morphology of a wild type control grown under these conditions is shown in Figure 1. (**C**) Mature hyphae stained with FM4-64 revealed similar polarity defects and the excessive formation of multiple septa in *cdc24(10-19)*, *cdc24(19-3)* and *cdc24(24-21)*. Arrows indicate FM4-64 accumulation in tips lacking a typical Spk.

The *Neurospora* genome project had generated a heterokaryotic deletion strain for the GEF *cdc-24*. However, homokaryotic knockout ascospores obtained by backcrossing with wild type only rarely germinated apolarly and ultimately lysed, indicating that *cdc-24* is essential for viability (Figure 4 A). Moreover, the phenotypic defects of the conditional *rac* and *cdc-42* mutants were highly reminiscent to conditional *cdc-24* mutants that also displayed multiple forms of apical hyperbranching and loss of polarity [25].

Of the ≥20 *cdc-24* strains isolated in this screen, we analyzed tree mutants that represented weak, intermediate and strong *cdc-24* defects (Figure 4 B, C). After shift to restrictive conditions, *cdc-24(10-19)* displayed pronounced apical hyperbranching, resulting in the formation of dense tree-like hyphal structures that looked highly similar to the *rac(7-1)* characteristics. Confocal imaging at higher magnification revealed distorted, but still polarly growing hyphae without the characteristic internal cell organization, i.e. hyphae showed lack of typical Spk, nuclei were closer to tips and no elongated mitochondria were observed (Figure 4 C). Severe morphological defects were observed in *cdc-24(19-3)* and *cdc-24(24-21)*, where hyphae branched excessively in apical and subapical regions. This was accompanied by swelling of apical tips and hyperseptated chains of cells without internal organization (Figure 4 C). Hyphal tips lost polarity altogether and ballooned spherically. At later stages hyperseptated hyphae resembled chains of spheres as apical and subapical hyphal compartments expanded isotropically, with some lysing at an advanced stage. The defects of these strains were almost identical to those of the conditional *rac(7-1);cdc-42(18-4)* double mutant described above. When conidia of the three *cdc-24* mutants were germinated at 37°C, polarity defects with similar characteristics were observed. *Cdc-24(10-19)* formed hyperbranched and tight colonies, while similar hyperbranching was accompanied by apical and subapical swelling of hyphal compartments in *cdc-24(19-3)*. *cdc-24(24-21)* conidia were unable to polarize and growth was restricted to isotropic expansion. These data underline the importance of CDC-24 not only for maintenance, but also for establishing cell polarity in *N. crassa*.

**Figure 5:**
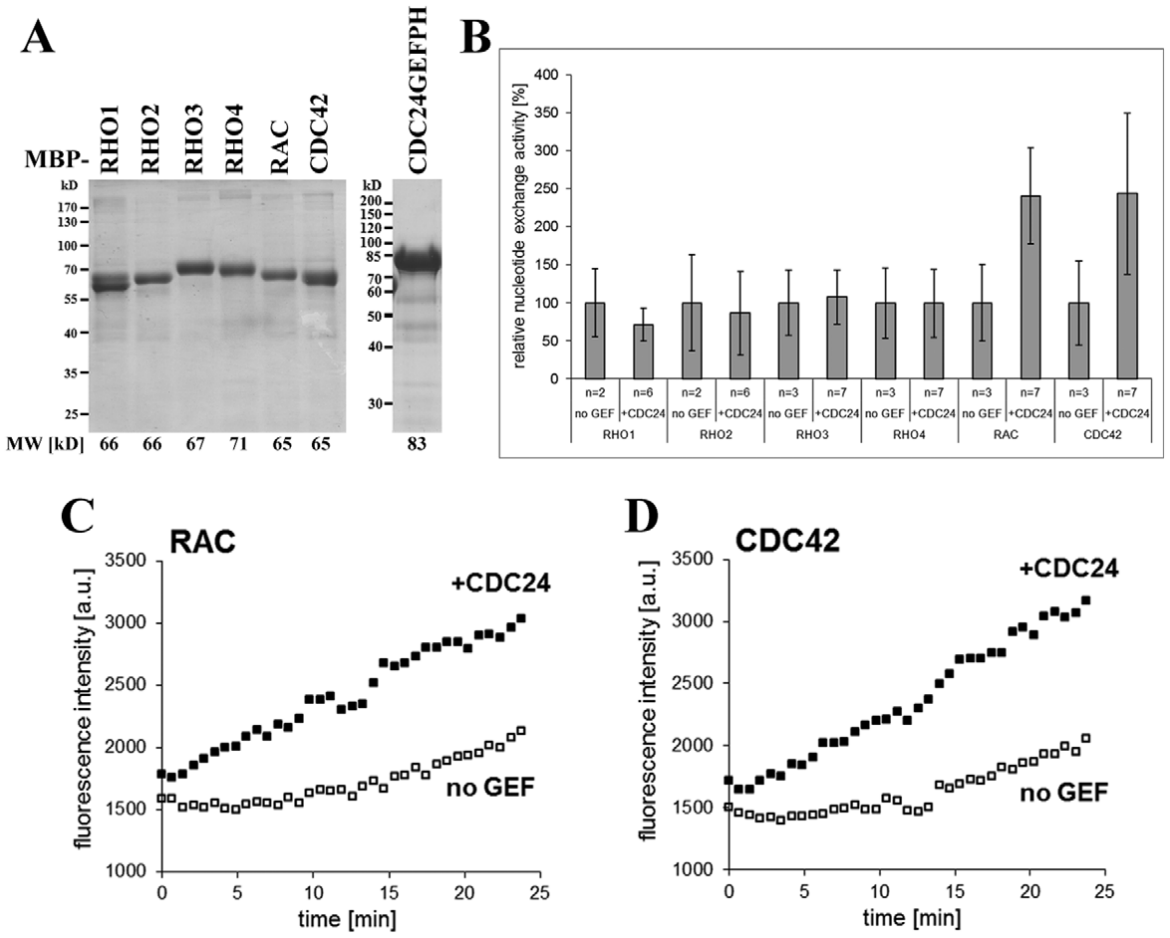
CDC24 functions as exchange factor for RAC and CDC42 *in vitro*. (**A**) Result of a representative purification of MBP-Rho GTPase fusion proteins and the MBP-CDC24-GEFPH construct used in *in vitro* GEF activity assays. A Coomassie Blue stained SDS polyacrylamide gel loaded with equal volumes of eluate fractions of the indicated constructs is shown. Predicted fusion protein molecular weights (MW) are given below the corresponding lanes. (**B**) Nucleotide exchange activity is displayed normalized to the intrinsic exchange activity (“no GEF”) of each Rho GTPase (set to 100%). n = number of independent experimental replicates, each of which was performed in technical duplicates. Error bars indicate standard deviation. (**C**) Kinetics of fluorescence emission intensity [a.u. = arbitrary units] plotted over time for representative individual experiments testing the nucleotide exchange activity of RAC and CDC42 in the absence (“no GEF”) or presence (“+CDC24”) of MBP-CDC24GEFPH.

Therefore, we determined the specificity of CDC-24 for its cognate *N. crassa* Rho GTPase(s) and performed *in vitro* GDP-GTP exchange assays with bacterially expressed and purified Rho proteins and a CDC-24 fragment that contained the catalytic GEF and adjacent PH domain of CDC-24 (Figure 5). CDC-24(204-544) specifically stimulated the GDP-GTP exchange activity of RAC and of CDC-42, but did not affect the exchange activity of RHO1 to RHO4. Interestingly, this fragment exhibited equal GEF activity towards RAC and CDC-42.

**Figure 6:**
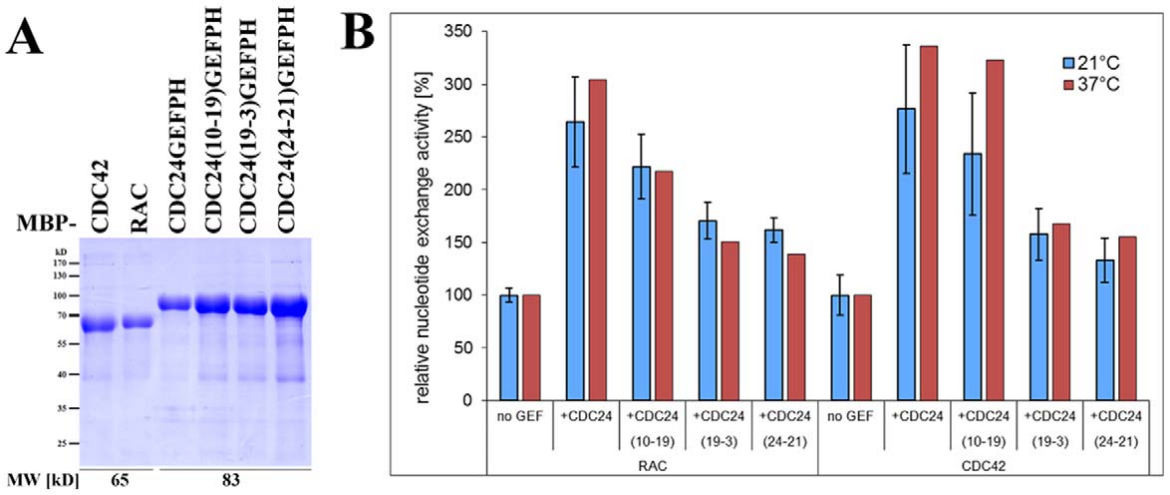
GTPase specificity is not altered in the mutant CDC-24 variants. (**A**) Purification of mutant MBP-CDC24-GEFPH constructs used in *in vitro* GEF activity assays. A Coomassie stained SDS polyacrylamide gel loaded with equal volumes of eluate fractions of the indicated constructs is shown. Predicted fusion protein molecular weights (MW) are given below the corresponding lanes. (**B**) Mutant RhoGEF-PH fragments exhibit reduced *in vitro* GEF activity towards both RAC and CDC42 compared to the wild type construct irrespective of the temperature used for the assay. Nucleotide exchange activity is displayed normalized to the intrinsic exchange activity (“no GEF”) of each Rho GTPase (set to 100%). Data from two independent experiments (with two technical replicates of each sample) were performed for 21°C (blue columns); results are based on one experiment performed in technical triplicates; for 37°C (red columns). Error bars indicate standard deviation.

Next, we asked if this dual GTPase specificity is affected in the conditional *cdc-24* mutants. Sequence analysis of the mutant *cdc-24* genes revealed mutations causing substitutions of highly conserved amino acids located within the predicted RhoGEF domain (*cdc-24(10-19)* and *cdc-24(24-21)*) or the adjacent PH domain (*cdc-24(19-3)*) of CDC-24 (Figure S2). Partial cDNA encoding the GEF and PH domains of CDC-24 (aa 204-544) was prepared from the three mutant strains, and the bacterially expressed proteins were used for *in vitro* GEF assays (Figure 6). The identified amino acid substitutions in the CDC-24 mutant constructs affected their ability to enhance nucleotide exchange in RAC and CDC-42, and the reduction in GEF competency of the mutant proteins correlated with the strength of morphological defects observed in the corresponding mutant strains. However, the three CDC-24 variants did not exhibit significantly altered target specificity *in vitro* when assayed at permissive or restrictive temperature.

### CDC-42, RAC and CDC-24 show distinct localizations patterns during cell polarization and tip extension

**Figure 7:**
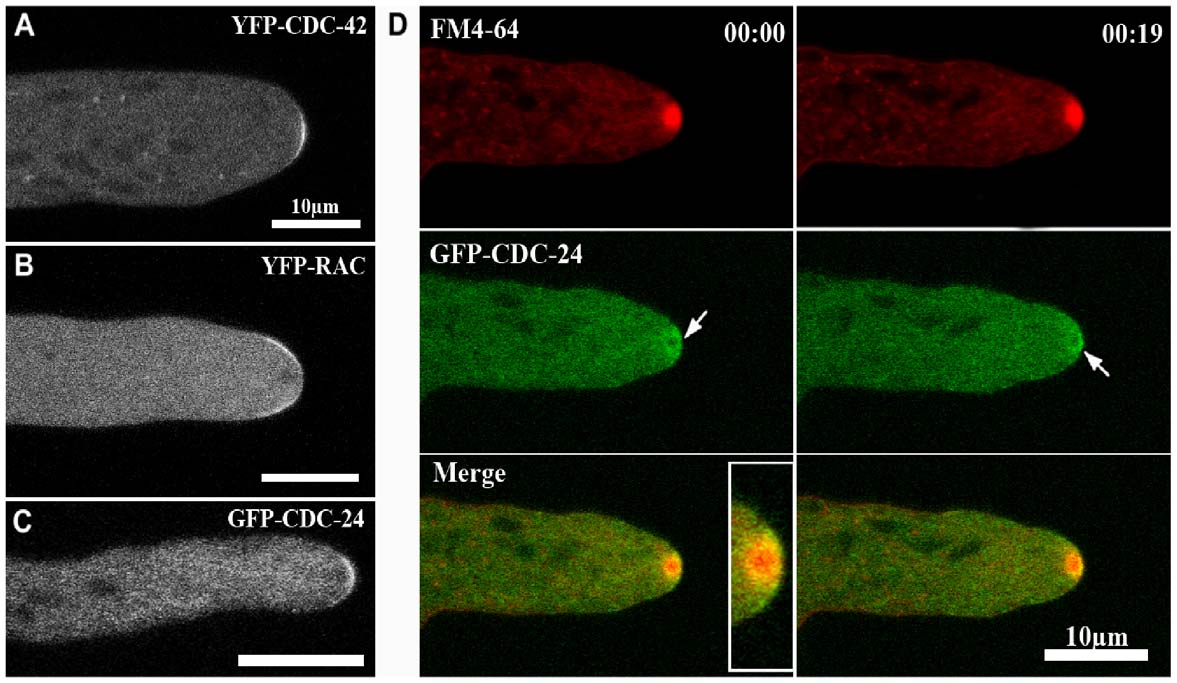
CDC-42, RAC and CDC-24 display partially distinct localization patterns in mature hyphal tips. Confocal laser scanning confocal microscopy of functional N-terminal fusion constructs of YFP-CDC-42 (**A**), YFP-RAC (**B**) and GFP-CDC-24 (**C**) reveal distinct localizations. CDC-42 localized as confined apical membrane-associated crescent, while RAC labeled a membrane-associated ring excluding the region labeled by CDC42. The GEF CDC-24 occupied a strategic position at the apical dome and localized as broad apical crescent covering the localization pattern of both GTPases and the apical cytosol. (**D**) Time course of GFP-CDC-24 co-localization with the vital dye FM4-64. Arrows indicate a dynamic accumulation of cytosolic CDC-24, clearly excluding the Spk core.

**Figure 8:**
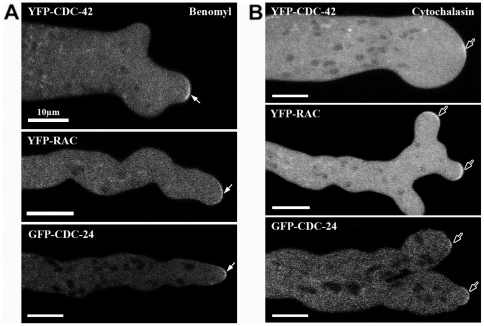
The membrane association of CDC-42, RAC and CDC-24 is not affected by disrupting the actin or microtubule cytoskeleton. Confocal images of vegetative hyphae treated with 2.5 µg/ml of the anti-microtubule drug benomyl (**A**) and 1 µg/ml f-actin inhibitor cytochalasin A (**B**). Note the distorted hyphal morphology caused by the two inhibitors; arrows point at YFP-CDC-42, YFP-RAC and GFP-CDC-24 at the abnormally shaped apical tips.

**Figure 9:**
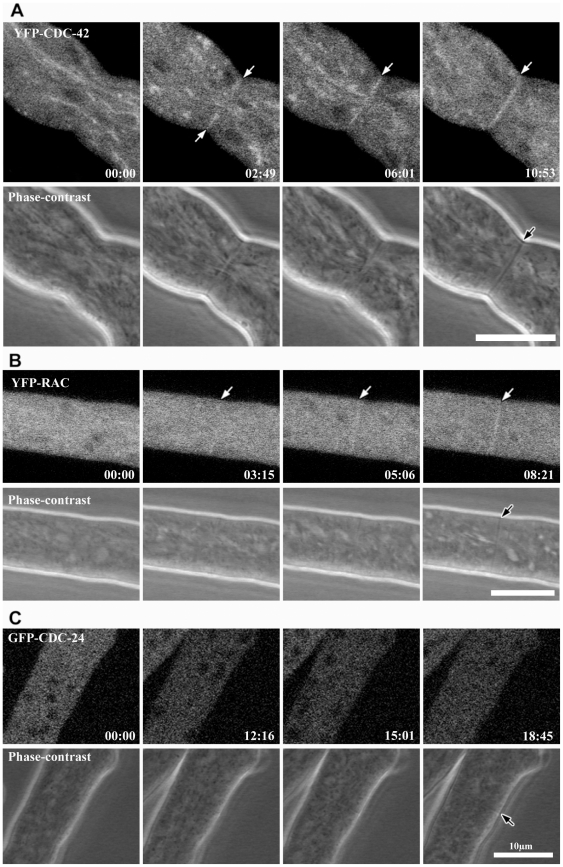
CDC-42 and RAC localize to forming septa. Time series of septum constriction in hyphae labeled with YFP-CDC-42 (**A**), YFP-RAC (**B**) and GFP-CDC-24 (**C**). White arrows indicate septum development detected in the fluorescence channel, and black arrows indicate the corresponding septum in the phase-contrast image.

*N. crassa* strains, in which CDC-42 and RAC GTPases were N-terminally tagged with YFP in the respective deletion background, complemented the mutant growth defects, indicating functionality of the constructs. We observed YFP-CDC-42 fluorescence in growing hyphal tips in the form of a plasma membrane-associated crescent by confocal microscopy (Figure 7 A, movie S1). In contrast, YFP-RAC localized as membrane-associated ring that excluded the most apical zone occupied by the Spk, which is labeled by YFP-CDC-42 (Figure 7 B, movie S2). As expected by its dual function as GEF for RAC and CDC-42, the localization of an N-terminal GFP-CDC-24 construct overlapped with those of both GTPases in that it was distributed as a broad cap at the hyphal apex (Figure 7 C). Interestingly, GFP-CDC-24 was not exclusively associated with the apical membrane as the two GTPases did, but also labeled a cytosolic region surrounding the Spk in a highly dynamic manner (Figure 7 D; movie S3). Counter-staining with FM4-64 further revealed that CDC-24 was excluded from the Spk core. In summary, the three components of the RAC – CDC-42 – CDC-24 GTPase module displayed distinct localization patterns within the mature hyphal tip. To explore if the three components of the GTPase module localize to the apical dome in a cytoskeleton-dependent manner, mature hyphae were exposed to cytochalasin A, which depolymerizes actin filaments and benomyl, a microtubule depolymerizing drug. When exposed to either drug, the three proteins remained associated with the apex despite the clear effect of the drugs, which provoked irregular hyphal growth and loss of polarity, respectively (Figure 8 A, B). These results indicate that the preservation of the three proteins at the hyphal tip is independent of a functional F-actin and microtubule cytoskeleton. Both GTPases were also observed at developing septa (Figure 9), but we did not detect CDC-24 there, potentially because the localization of all three proteins at septa is very weak and close to the detection limit.

**Figure 10:**
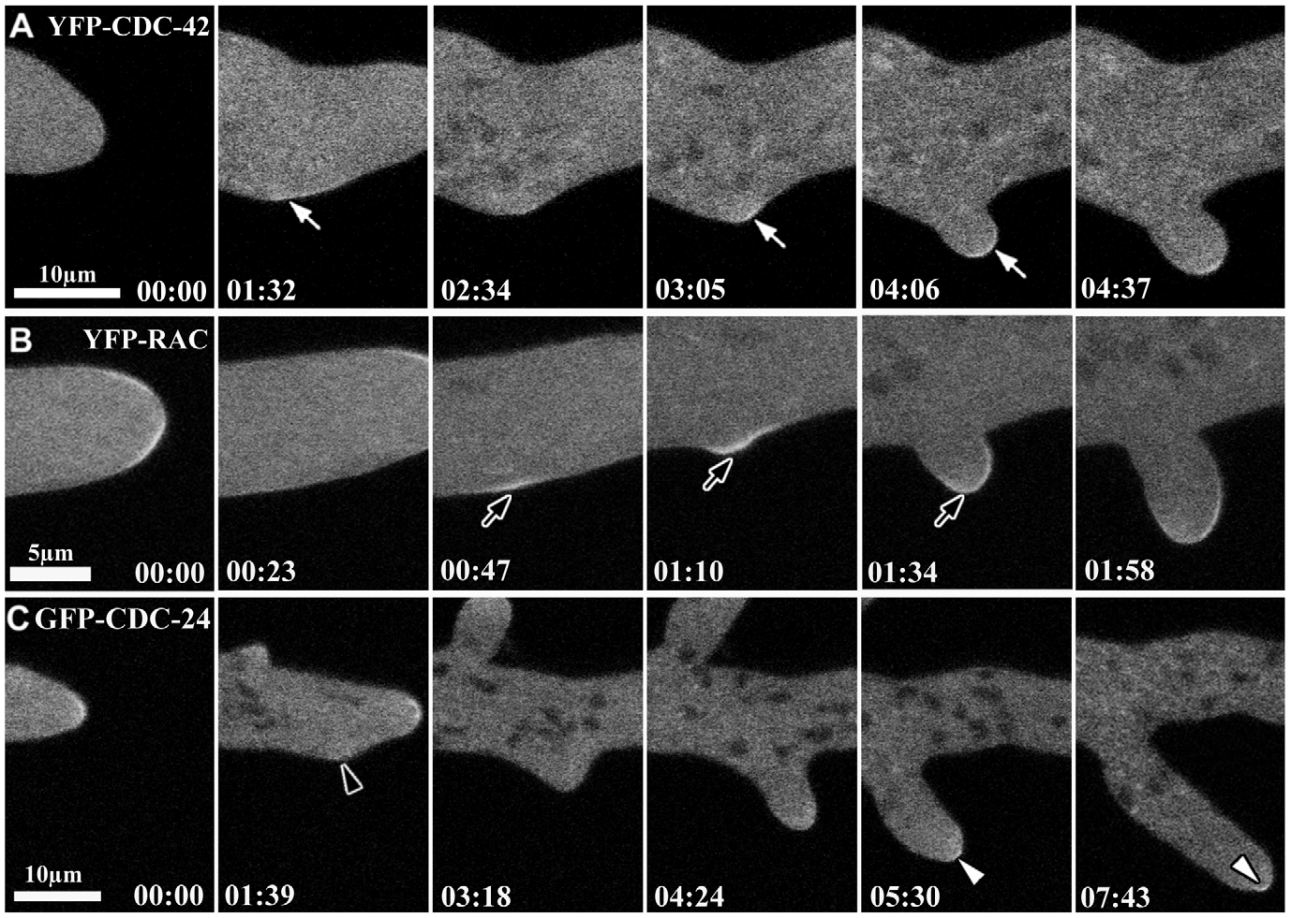
CDC-42 and RAC participate in lateral branch initiation. Time series taken by laser scanning confocal microscopy of YFP-CDC-42, YFP-RAC and GFP-CDC-24 during lateral branching emergence. (**A–B**) Accumulation of CDC-42 and RAC at subapical region of the plasma membrane prior to the emergence of the new branch and after of establishment of a new axis of polarity are indicated by white and black arrows, respectively. (**C**) CDC-24 accumulation was not detected close to the plasma membrane (black arrowhead), but only as a cytoplasmic cloud, occupying the apical dome in the new branch as indicate the white arrowheads.

The phenotypic characteristics of *rac* and *cdc-42* mutants indicated the involvement of these GTPases not only in hyphal growth, but also in polarity establishment of a germinating spore and during branch formation. We observed a slight accumulation of both proteins at a subapical region of the plasma membrane, ca. 20–70 sec prior to the emergence of the new branch (Figure 10 A, B), further supporting the involvement of CDC-42 and RAC in the polarity establishment. Interestingly, CDC42 accumulated at future branch sites ≥1 min prior to branch emergence, while RAC localized there only ≤20 sec. Both proteins maintained their localization within the apex of newly formed branches with a very dynamic behavior (movies S4, S5). Interestingly, RAC was observed as crescent throughout the whole apical dome of newly formed branches (Figure 10 B, movie S5), a different distribution pattern than that observed in mature hyphae and more similar to the localization of RAC in the germ tube (see below), suggesting a growth rate dependent re-localization of RAC from an apical crescent to a subapical ring. Once, the new branch reached a length of about 20–30 µm, RAC adopted a subapical distribution as observed in mature hyphae. CDC-42 also displayed a wider distribution at the apex of new branches, compared to that of leading hyphae (movie S4). In contrast, CDC-24 was not detected as an accumulation close to the plasma membrane, but only as a cloud occupying the apical dome (Figure 10 C, movie S6). A slight accumulation in the tip was detected once the new branch reached approximately 10 µm in length (Figure 10 C).

**Figure 11:**
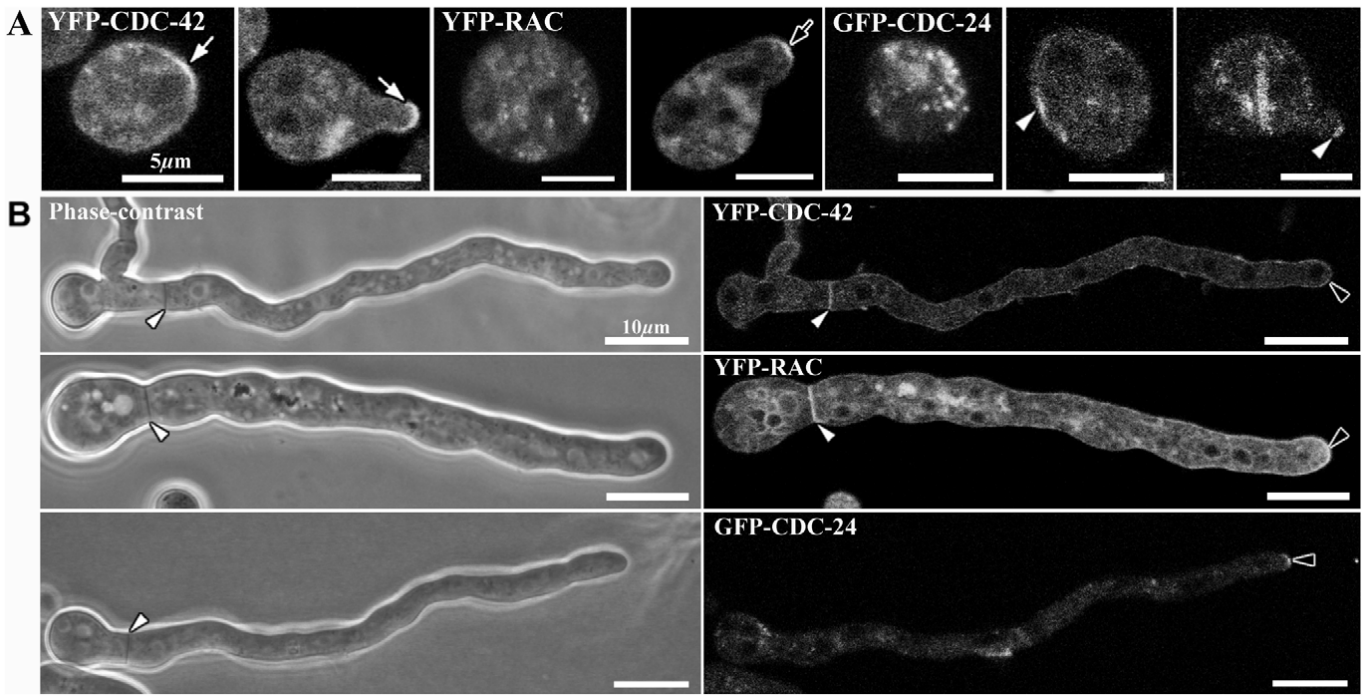
CDC-42, RAC and CDC-24 display distinct localization patterns during germination. (**A**) Fluorescent images show the localization of YFP-CDC-42, YFP-RAC and GFP-CDC-24 during spore germination and germ tube emergence. White arrows indicate the presence of YFP-CDC-42 at discrete region of the spore indicated a new polarization site and indicate that CDC-42 continues present at apical dome during germ tube emergence. Black arrow indicates the localization of YFP-RAC during germ tube elongation. White arrowhead indicates the localization of GFP-CDC-24 at discrete region of the spore and germ tube elongation. (**B**) Phase-contrast and Fluorescent images showing the localization of YFP-CDC-42, YFP-RAC and GFP-CDC-24 in germlings of *N. crassa*. Note that CDC-42 and RAC are present in the septum development in germlings, but CDC-24 was not detected in this process. White arrowheads show septum formation by phase-contrast and fluorescent microscopy. Black arrowhead indicates the localization of YFP-CDC-42, YFP-RAC and GFP-CDC-24 at the apex of germlings.

When conidia (asexual spores) are transferred to an appropriate medium, they rehydrate and begin to grow isotropically for 3–4 h, before growth becomes polarized, and a new hyphal tip is generated [4], [8]. During this hydration phase, YFP-CDC-42 accumulated at a discrete zone of the conidia that marked the future site of germ tube emergence, while the YFP-RAC was observed as cytosolic spots and accumulated at the membrane only after polarization had occurred, labeling the apex of an established germ tube (Figure 11 A). GFP-CDC-24 was primarily observed as cytosolic spots and less frequently accumulated at the cortex of conidia. Its accumulation at the apex of germ tubes was typically visible only after the germ tube reached a few µm in length. During extended growth of the germling, both YFP-CDC-42 and YFP-RAC accumulated as general plasma membrane label with an increased accumulation within the apical 5–10 µm (Figure 11 B). In contrast to RAC and CDC-42, CDC-24 appears to accumulate as a cloud occupying the apical region, before its membrane association became stronger with increased growth rate of the hyphal tip.

## Discussion

The results presented here show that the RAC – CDC-42 – CDC-24 GTPase module is required for polarized growth and hyphal morphogenesis in the ascomycete *N. crassa*. The phenotypic characterization of single and double mutants and a thorough microscopic analysis of the localization patterns of the three proteins indicate that the two Rho GTPases have primarily non-redundant functions. However, they must at least share one common and essential task during establishment and maintenance of cell polarity, which is illustrated by the synthetic lethality of *rac;cdc-42* double mutants. Moreover, *in vitro* GDP-GTP exchange assays demonstrate that CDC-24 functions as common GEF for RAC and CDC-42 and the mutant characteristics of conditional as well as loss of function alleles of *rac*, *cdc-42* and *cdc-24* strongly suggest that CDC-24 is the primary GEF for RAC and CDC-42.

Cells devoid of either of the two GTPases are still able to germinate, but show clear growth and polarity defects that result in the formation of small compact colonies. This signifies that establishment of a primary axis of polarity is possible, albeit delayed, in the absence of CDC-42 or RAC, but subsequent hyphal extension is highly compromised in distinct ways. Strains deficient in RAC are characterized by dichotomous tip splitting and massive apical hyperbranching; this is also observed to a lower extent in mutants affected in CDC-42 function, but their most prominent feature is the emergence of numerous subapical branches and the swelling of apical and subapical regions of the hypha. Thus, the two GTPases appear to function jointly in establishing polarity and in maintaining a stable axis of polarity, with a greater impact of RAC on the latter process, whereas CDC-42 is required to control the overall cell morphology and subapical branching. This notion of both overlapping and individual roles of the two GTPases in hyphal morphogenesis is corroborated by the findings that the simultaneous depletion of RAC and CDC-42 is synthetically lethal and a conditional double mutant results in complete loss of polarity.

In budding and fission yeasts, Cdc42p activity is regulated by the RhoGEF Cdc24p or its close homologue Scd1, respectively [11], [12]. Cdc24 have also been implicated in the regulation of Cdc42 in *A. gossypii* and *C. albicans* [18], [41]. However, for none of the species GEF activity of Cdc24 towards the presumed target GTPase has been directly demonstrated. In contrast, *U. maydis* Cdc24 functions as specific activator of Rac1 [13], [42], [43]. Thus, this study presents the first evidence that CDC-24 stimulates *in vitro* nucleotide exchange of both RAC and CDC-42 in a fungal system. Consistent with these *in vitro* results, the polarity defects observed in conditional *cdc-24* mutants phenocopy those observed for mutants deficient in RAC and CDC-42 function. *cdc-24(10-19)* and *cdc-24(19-3)* exhibit clear apical hyperbranching as determined for *rac(7-1)* and, less pronounced, for *cdc-42(18-4)*, while the phenotypic characteristics of *cdc-24(24-21)* are identical to that of the conditional *rac(7-1)*;*cdc-42(18-4)* double mutant. Specifically, *cdc-24(24-21)* and *rac(7-1)*;*cdc-42(18-4)* conidia are unable to perform the isotropic to polar growth switch required for spore germination, and ascospores homokaryotic for deletion of *cdc-24* and *rac*;*cdc-42* fail to establish polarity. Moreover, when established colonies of the two conditional strains are transferred to restrictive condition, the hyphae lose polarity, hyper-branch and continue growing in an isotropic manner.

**Figure 12:**
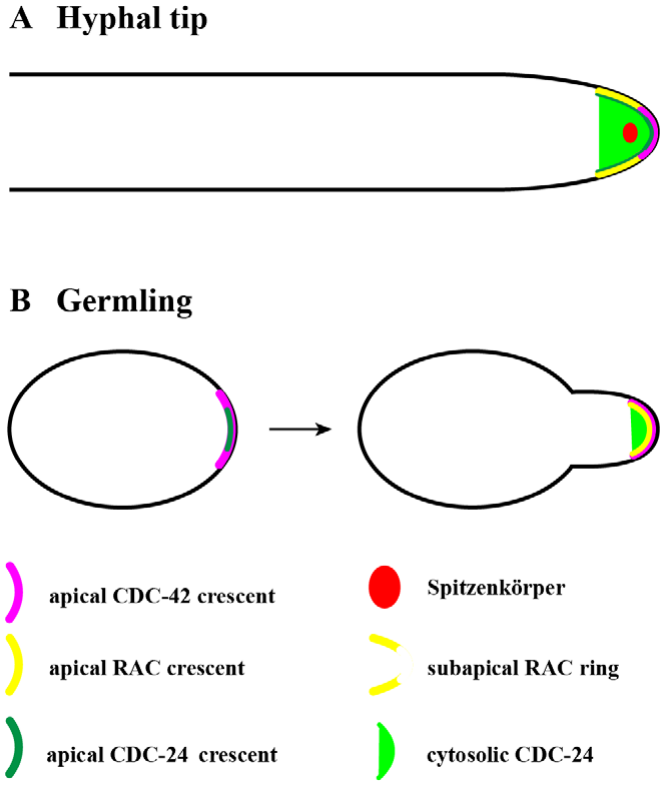
Model representing the localization of the CDC-42 – RAC – CDC-24 module in *Neurospora crassa*. Schematic summary of the localization patterns of CDC-42, RAC and CDC-24 in growing hyphal tips (**A**) and during germination (**B**).

The proposed functional overlap of RAC and CDC-42 in *N. crassa* and their common regulation by CDC-24 is also reflected in the similar localization patterns of the three proteins. Both GTPases are concentrated as membrane-associated crescent at sites of polarization during germ tube and branch formation, the hyphal apex of mature hyphae and at constricting septa. Accumulation of Rac and Cdc42 homologues at hyphal tips, often in crescent-like structures as observed in this study, has been reported for several filamentous fungi such as *P. marneffei*, *A. nidulans*, *A. niger* and *C. albicans* [14], [22], [24], [44]–[46] and is further underlining the importance of the two GTPases for fungal morphogenesis. Interestingly, the specific localization patterns of the three proteins are distinct, and support different functions of RAC and CDC-42 within the mature hyphal tip and during polarity establishment. CDC-42 localized as confined apical membrane-associated crescent in the hyphal tip, while RAC labeled a membrane-associated ring excluding the region labeled by CDC42 (Figure 12 A). The GEF CDC-24 occupies a strategic position at the apical dome, localizing as broad apical crescent covering the localization pattern of both GTPases. This is consistent with the *in vitro* GDP-GTP exchange assays that confirm equal GEF activity towards RAC and CDC-42. However, CDC-24 also displays a cytosolic accumulation surrounding the Spk, suggesting that activation of RAC and CDC-42 occurs at the plasma membrane, while cytosolic CDC-24 may serve as activation competent reservoir or may have additional GEF-independent functions.

The localization of the two GTPases in young germlings and in newly established branches is different from that observed in mature hyphae, potentially because the two GTPases re-localize in a growth rate dependent manner at the hyphal tip, similarly to what has been described for the RHO-1 GAP LRG-1 in *N. crassa* [26]. Specifically, both CDC-42 and RAC localize in germlings and during the formation of new branches as broad membrane-associated crescents within the apical dome and switch to a small apical cap and a subapical ring, respectively, once tip extension has reached a certain rate. Cdc42 has already been implicated in branch formation in *A. nidulans* [14], although convincing data are currently lacking. In *N. crassa*, CDC-42 and RAC localize to future branch points prior to their emergence, implicating both proteins in the regulation of branch initiation. However, both proteins are not essential for branch formation as both deletion strains are still able to branch. Interestingly, the two GTPases have different kinetics of membrane localization prior to branch emergence, suggesting early and late functions of CDC-42 and RAC, respectively during branch initiation. Even more pronounced is the difference in localization of the two GTPases during polarity establishment in conidiospores (Figure 12 B): while CDC-42 localizes to the cortex prior to germ tube emergence, RAC accumulates there only after polarity is established. These differences correlate with a more pronounced polarity defect of Δ*cdc-42* compared to Δ*rac* and may suggest a more important role of CDC-42 than RAC during polarity establishment in *N. crassa*.

CDC-42 and RAC also participate in septum formation, consistent with a function described for Cdc42p in budding and fission yeasts during cell division [11], [12]. Likewise, septal localization has also been observed for CflA and CflB in *P. marneffei*, where loss of the latter leads to inappropriate septation [44], [45], for Cdc42 in *C. albicans* [22] and, rarely, for RacA in *A. niger* [24]. With the exception of *U. maydis* Cdc42, which appears to be highly specialized for controlling cell separation of the yeast form of this basidiomycete fungus [47], specific contributions of Rac and Cdc42 during septum formation in filamentous fungi remain to be elucidated. The increased abundance of septa in the *N. crassa rac*, *cdc-42*, and *cdc-24* mutants suggests an involvement as negative regulators that may function in an antagonistic relationship with the GTPases RHO1 and RHO4, which are positive regulators of septum formation in *N. crassa*, *A. nidulans* and *C. albicans* [26], [35], [48]–[51].

While a more detailed analysis of the localization kinetics and activation patterns of RAC – CDC42 – CDC-24 module in *N. crassa* essential for their mechanistic understanding, the identification of shared and unique downstream effector proteins is also required for clarifying their common and distinct roles during hyphal morphogenesis. Potential effectors include the PAK family kinases Cla4 and Ste20 that function during actin organization, MAP kinase activation and septin organization and have been implicated as RAC/Cdc42 targets in various filamentous fungi [43], [52]. Other potential effectors are components of the ROS production machinery, which have been implicated in regulating apical dominance of fungal hyphae [53], [54] and shown to interact with RAC in *A. niger* and *Epichloë festucae* [24], [55]. In light of the multitude of morphogenetic factors possibly acting downstream of RAC and CDC42, much additional work is needed to elucidate the common and individual output pathways, which ultimately determine the pattern of redundancy and specialization observed for the two GTPases in *N. crassa* and other filamentous fungi.

## Supporting Information

Figure S1 **Sequence alignments of fungal RAC and CDC-24 homologs.** Amino acid substitutions of the conditional *cdc-42* (**A**) and *rac* (**B**) mutants are highlighted.
(TIF)

Figure S2 **Sequence alignments of fungal CDC-24 homologs.** Amino acid substitutions of the conditional *cdc-24* mutants are highlighted.
(TIF)

Movie S1 **Dynamic distribution of YFP-CDC-42 in mature hyphal tips.**
(MP4)

Movie S2 **Dynamic distribution of YFP-RAC in mature hyphal tips.**
(MP4)

Movie S3 **Dynamic distribution of GFP-CDC-24 in mature hyphal tips.**
(MP4)

Movie S4 **Time-course of YFP-CDC-42 localization during lateral branch initiation.**
(MP4)

Movie S5 **Time-course of YFP-RAC localization during lateral branch initiation.**
(MP4)

Movie S6 **Time-course of GFP-CDC-24 localization during lateral branch initiation.**
(MP4)

Table S1 **Plasmids used or generated in this study.**
(TIF)

Table S2 **Oligonucleotides used in this study.** Restriction endonuclease sites for cloning are shown in bold type and the start codon is underlined.
(TIF)
