# Supplementary figures and images for: Functional Characterization and Cellular Dynamics of the CDC-42 – RAC – CDC-24 Module in Neurospora crassa
Source: PLoS One. 2011 Nov 7;6(11):e27148. doi: 10.1371/journal.pone.0027148 (PMC3210136; doi:10.1371/journal.pone.0027148)

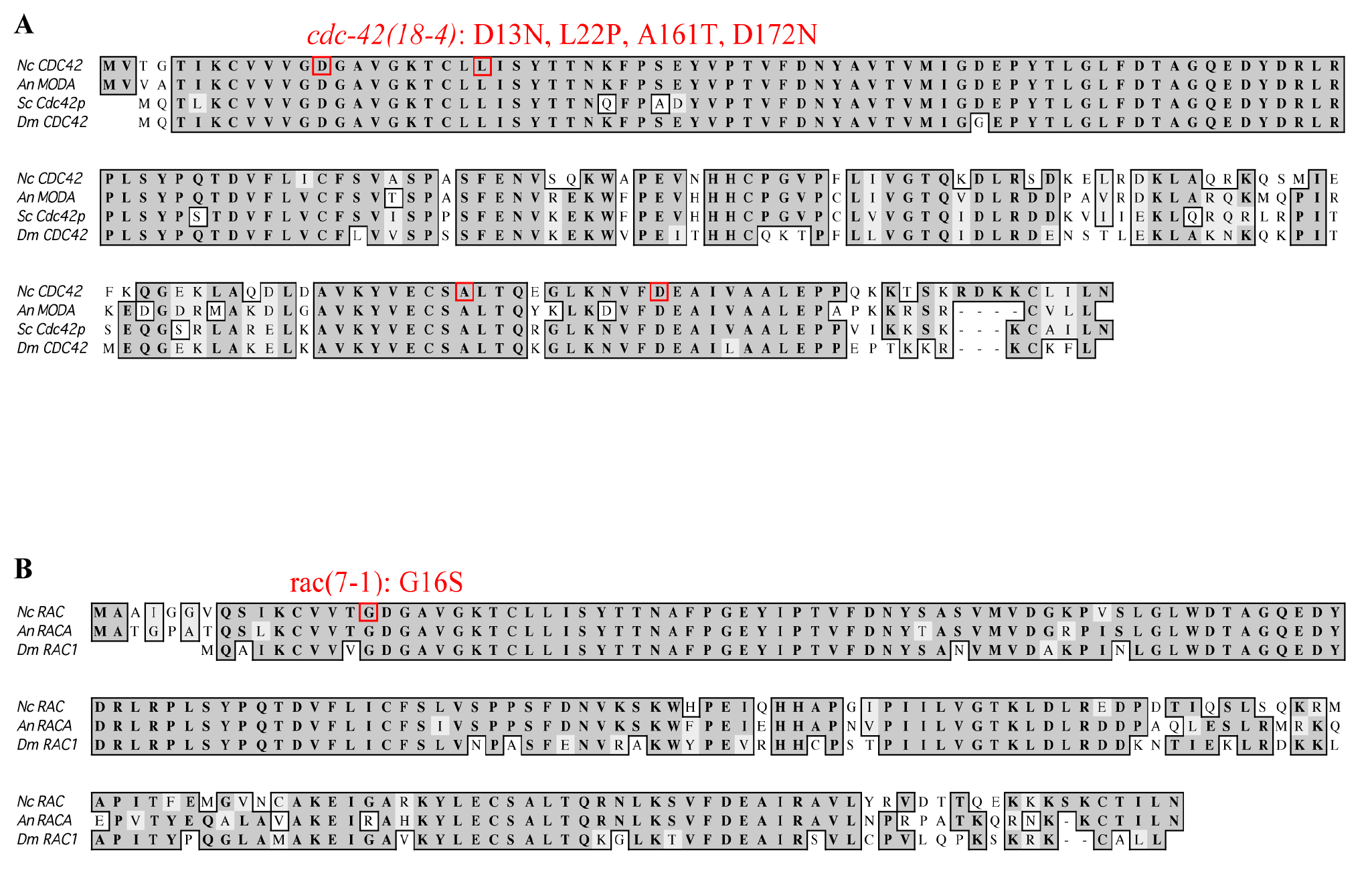

Supplement: Figure S1 — Sequence alignments of fungal RAC and CDC-24 homologs. Amino acid substitutions of the conditional cdc-42 (A) and rac (B) mutants are highlighted. (TIF) [file pone.0027148.s001.tif]

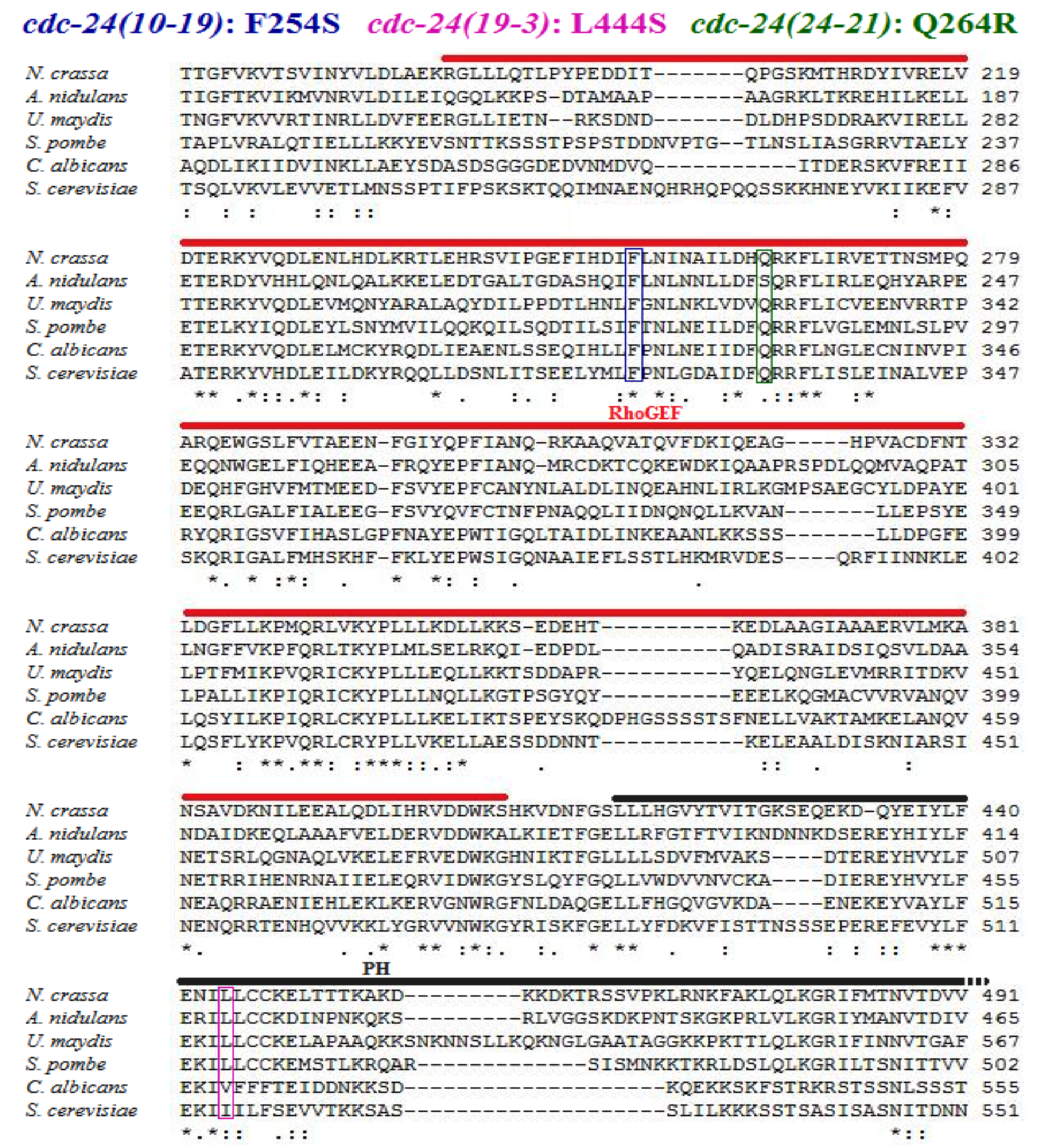

Supplement: Figure S2 — Sequence alignments of fungal CDC-24 homologs. Amino acid substitutions of the conditional cdc-24 mutants are highlighted. (TIF) [file pone.0027148.s002.tif]

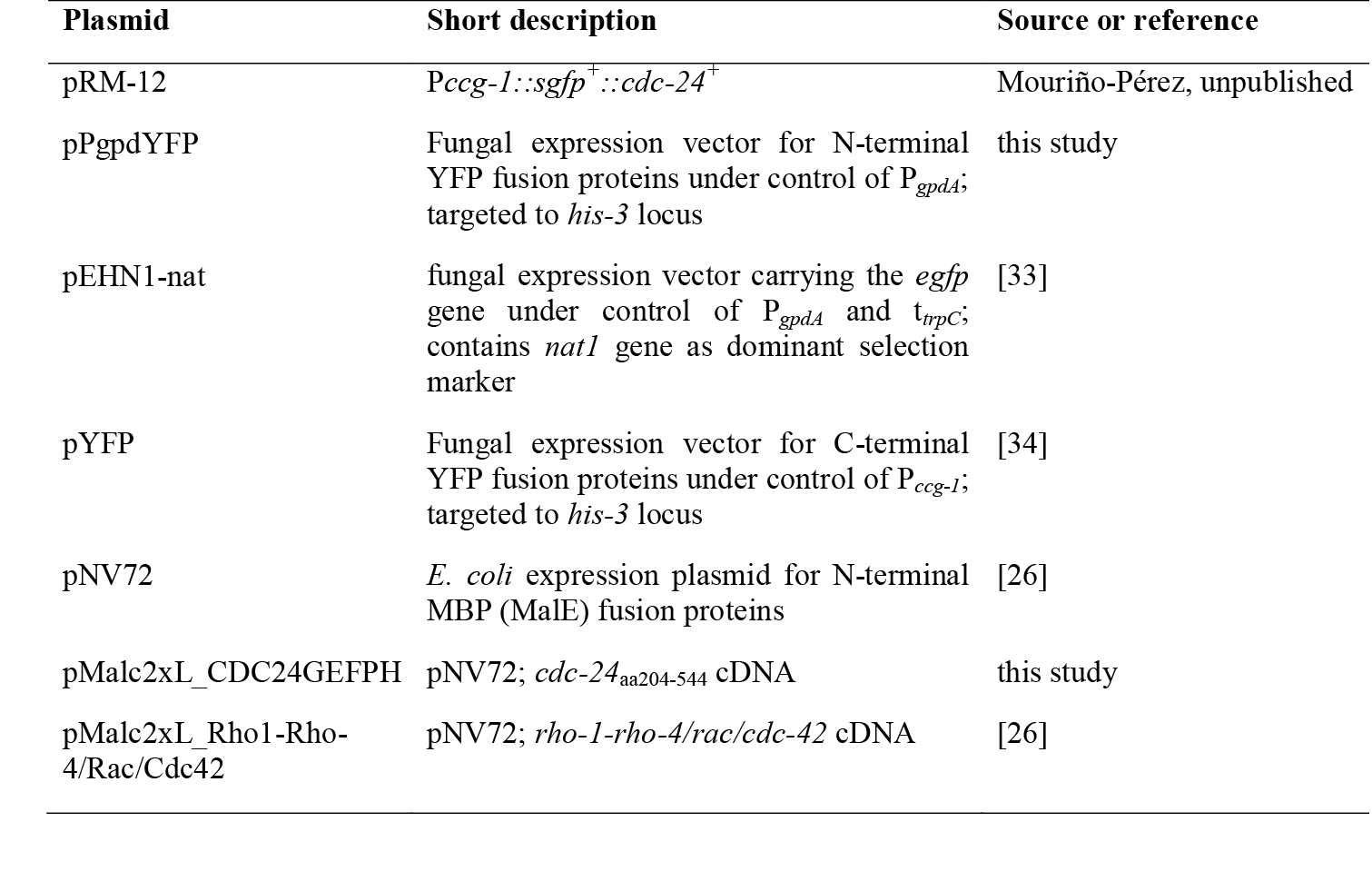

Supplement: Table S1 — Plasmids used or generated in this study. (TIF) [file pone.0027148.s009.tif]

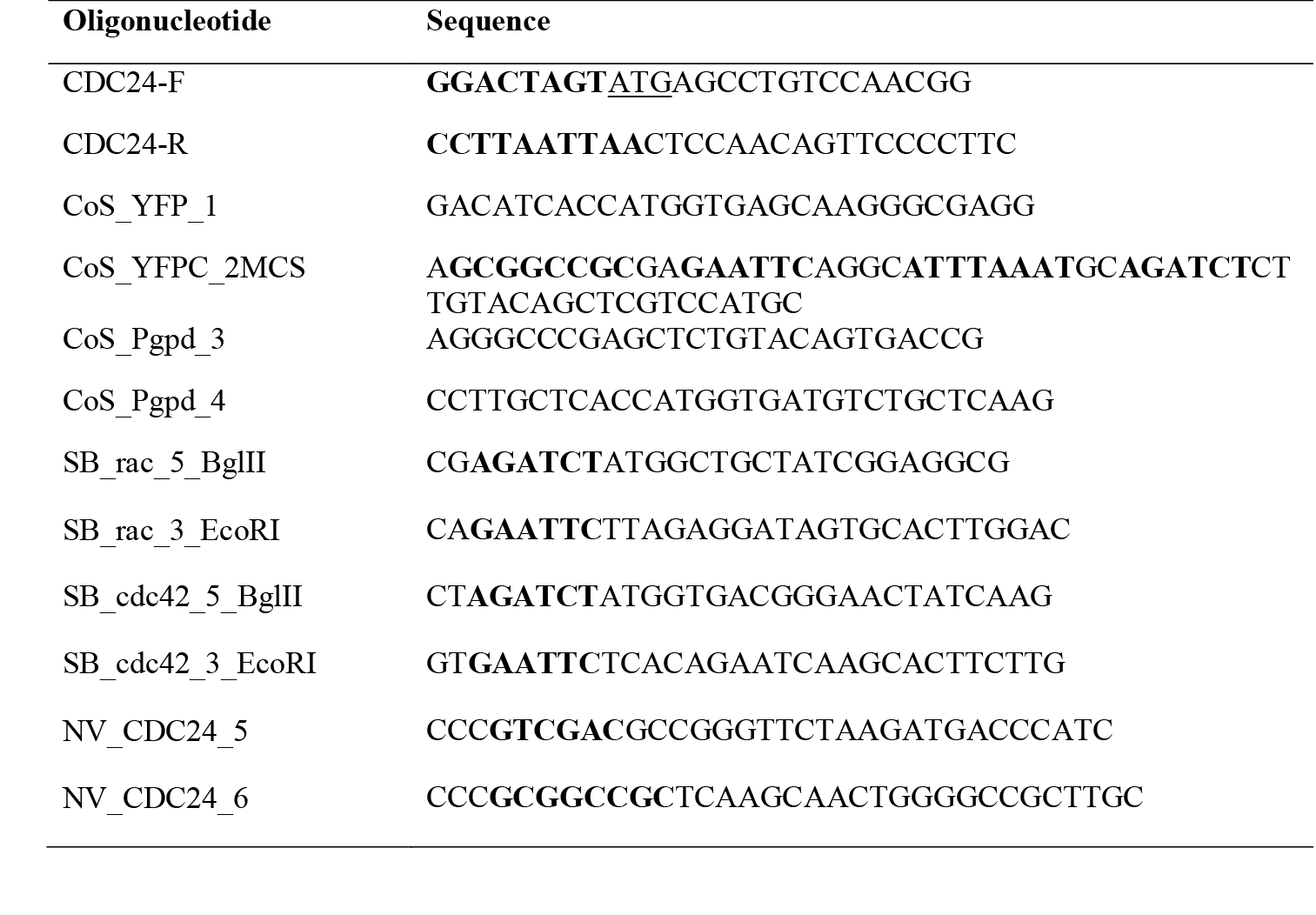

Supplement: Table S2 — Oligonucleotides used in this study. Restriction endonuclease sites for cloning are shown in bold type and the start codon is underlined. (TIF) [file pone.0027148.s010.tif]
